# Feasibility and Acceptability of Useful Field of View Cognitive Training in Adolescent Athletes: Pilot Pre-Post Study

**DOI:** 10.2196/93778

**Published:** 2026-09-11

**Authors:** Elizabeth D Rosenthal, Tyler A Busch, Stacy J Suskauer, Beth S Slomine, Erika F Augustine, Emily R Akrong, Adrian M Svingos

**Affiliations:** 1 Brain Injury Clinical Research Center, Kennedy Krieger Institute, 716 N. Broadway, Baltimore, MD, 21205, United States, 1 443-923-7947; 2 Department of Psychology, The Graduate Center, CUNY, New York, NY, United States; 3 Department of Clinical and Health Psychology, University of Florida, Gainesville, FL, United States; 4 Department of Physical Medicine and Rehabilitation, Johns Hopkins Medicine, Baltimore, MD, United States; 5 Department of Psychiatry and Behavioral Sciences, Johns Hopkins Medicine, Baltimore, MD, United States; 6 Clinical Trials Unit, Kennedy Krieger Institute, Baltimore, MD, United States; 7 Department of Neurology, Johns Hopkins Medicine, Baltimore, MD, United States

**Keywords:** adolescent, athletes, cognitive training, visual perception, useful field of view

## Abstract

**Background:**

Training visual processing skills, a domain implicated in sport performance and injury risk, could reduce injuries among youth athletes. The useful field of view (UFOV) cognitive training program improves visual processing efficiency. While UFOV training has been extensively studied in aging populations and yields significant cognitive and functional benefits, it has yet to be examined in adolescents.

**Objective:**

The aim of this study is to preliminarily assess the feasibility and acceptability of UFOV training in youth and report on the magnitude of change in visual processing efficiency from pre- to posttraining.

**Methods:**

Six adolescent athletes (aged 14‐18 years) completed a 5-week adaptive, online, remote UFOV training program. The UFOV training program has a gamelike interface and increases in difficulty as participants accurately identify and locate targets that are rapidly displayed in the center and at the periphery of their screen amid distractors. Participants were assigned a total training dose of 250 levels across 5 weeks, with 5 scheduled sessions per week, each lasting approximately 25 minutes (equating to approximately 10.42 hours of assigned training time). Individuals met remotely with research staff weekly to receive coaching. We examined feasibility by calculating overall dose adherence (percentage of assigned levels completed) and regimen adherence (percentage of scheduled training sessions completed). Average overall acceptability, program usability, adoptability, and perceived benefit were calculated using responses to a posttraining survey, where item-level responses ranged from 1 to 7 on a Likert scale (responses greater than 4 reflected a view more positive than neutral toward the program). Participants completed the Double Decision Assessment pre- and posttraining to quantify the magnitude of change in visual processing efficiency.

**Results:**

In this pilot cohort, we observed strong dose (mean 95.33%, SD 11.43%) and regimen (mean 90.67%, SD 12.56%) adherence. Participants’ responses to the acceptability survey suggested positive views of the program overall (mean 5.47, SD 0.4) and in terms of usability (mean 5.69, SD 0.43), perceived benefit (mean 5.36, SD 0.69), and adoptability (mean 5.17, SD 0.61). All participants demonstrated improved visual processing efficiency from pre- to posttraining (mean difference of 168.67, SD 112.05 ms; Cohen *d*=1.51).

**Conclusions:**

Though preliminary, results suggest that UFOV training may be feasible and acceptable for adolescent athletes. All participants in this pilot study demonstrated improvements in visual processing efficiency after completing 5 weeks of training. Further research is needed to investigate the feasibility of implementing this training in other contexts, such as without research team support or in a group setting. Future work should also evaluate the extent to which UFOV training improves visual processing more than an active control intervention and whether visual processing improvements transfer to untrained skills to reduce the risk of sport-related injury.

## Introduction

Sport-related injuries are exceedingly common in adolescent athletes, with estimates of injury prevalence ranging from 34% to 65% [1]. Contact sports, such as soccer, basketball, and football, confer especially high injury risk [1-3], with lower-extremity orthopedic injuries and concussions among the most common injuries sustained by athletes [4]. These injuries can lead to missed practices and competitions, absenteeism from school and associated learning losses, decreased quality of life, and mental health consequences [5-7]. Therefore, preventing injury among youth athletes is pivotal.

To date, numerous strategies have been implemented to reduce sport-related injury risk in youth sports. These include personal protective equipment, such as mouth guards and eyewear in hockey, rule changes (eg, banning cross-checking in ice hockey, reducing collision practices in football, and limiting pitch counts in baseball), tailored training strategies, including neuromuscular training warm-ups and resistance training, and reducing sport specialization by promoting multisport participation [8-11]. However, it is also important to consider interventions that bolster intrinsic, individual factors to build resilience and reduce the risk of sport-related injuries.

Visual processing efficiency—the speed and accuracy with which an individual can perceive and process visual stimuli—represents a promising target for intervention, as various visual cognitive abilities have been implicated in sport performance and injury risk. For instance, better visual attention skills are associated with lower-extremity musculoskeletal injury risk in adolescent athletes [12]. Faster visual reaction times allow players to better see the game around them and make faster decisions [13]. Furthermore, strong visual attention abilities are thought to help athletes anticipate collisions on the field and reduce sport-related injuries, including concussions, with peripheral vision playing a key role in the processing of multiple visual stimuli simultaneously [14]. Therefore, weaker visual processing skills may make athletes more vulnerable to on-field collisions and injuries [14]. Crucially, visual processing efficiency is modifiable through cognitive training [15-20], and improving youth athletes’ visual abilities may reduce sport-related injury risk.

The useful field of view (UFOV) cognitive training program has been studied extensively in adults and has been shown to reliably improve visual processing efficiency [15-20]. UFOV training focuses on improving visual processing speed and strengthening rapid visual detection of stimuli amid irrelevant visual clutter in the center and at the periphery of the visual field [21]. Importantly, improvements in visual processing efficiency gained through UFOV training have been shown to transfer to real-world improvements in functional outcomes, such as activities of daily living in older adults [17,22-25]. Enhanced visual and cognitive performance from UFOV training also improves older adults’ driving performance and safety [17,26-29]. Critically, improvements in visual processing efficiency have been shown to persist for periods ranging from 2 to 10 years, indicating that UFOV cognitive training can have lasting impacts on visual processing and real-life skills [17,19,25-30].

Given the benefits of UFOV cognitive training on visual processing in older adults and the importance of visual processing efficiency in reducing sport-related injury risk in youth, we hypothesize that UFOV training could benefit adolescent athletes at high risk for concussion and musculoskeletal injuries. Notably, visual processing skills improve throughout childhood and begin to reach adult levels in adolescence [31]. Therefore, this developmental period is ideal for interventions aiming to improve visual processing efficiency with long-lasting impacts. Developmentally, adolescence represents a period of ongoing maturation and neuroplasticity in frontoparietal attentional networks, suggesting potential responsiveness to targeted cognitive training interventions [32-34]. This pilot study is the first to examine UFOV cognitive training in youth. Here, we preliminarily assess the feasibility and acceptability of a 5-week UFOV training program in adolescent athletes and report on the magnitude of change in visual processing efficiency from pre- to posttraining as a foundation for future trials designed to evaluate efficacy.

## Methods

### Overview

Participants were recruited from a prior research study (they had provided consent for future research contact) and from the community (via posted flyers and word of mouth). For inclusion, participants were required to be aged 14 to 18 years at enrollment, capable of independently using a computer or tablet, and English speaking. Participants were enrolled between June and August 2023. Six participants were contacted and screened for inclusion and were determined to be eligible for the present study; all provided informed consent and completed the study. Participants included 6 typically developing athletes aged 14 to 18 years (mean 16.35, SD 1.64 years), including 5 (83.33%) males. Two participants had a history of concussion, which they had fully recovered from at least 1 year prior to enrollment.

**Figure 1.**
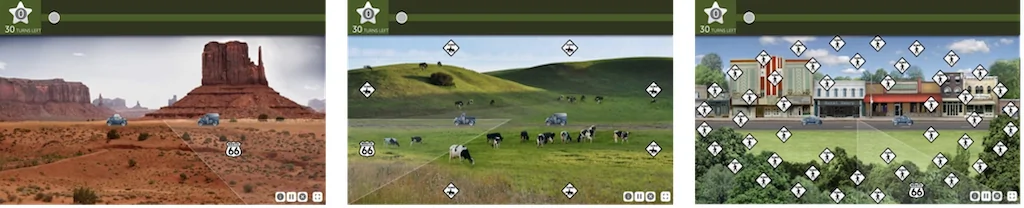
Example useful field of view (UFOV) training levels with varying difficulty: easy (left), medium (center), and hard (right). Images reproduced from BrainHQ with permission.

Each participant completed a 5-week adaptive UFOV training program using the Double Decision exercise [35], which increases in difficulty incrementally as performance improves and is designed to improve visual processing efficiency. The training program consisted of 5 remote, online sessions per week, each lasting approximately 25 minutes and consisting of 10 training levels. The overall assigned training dose was 250 levels, equating to approximately 10.42 total training hours per participant. Training levels involved a series of trials in which participants had to identify and locate targets that were rapidly displayed in the center and at the periphery of their screen amid distractors (Figure 1). A dose of 250 levels (approximately 10.42 total training hours) was selected because (1) available data in adults suggest that 10 or more hours of UFOV training will result in improved visual processing efficiency that will transfer to untrained skills [17] and (2) available data suggest that this amount of computerized cognitive training would be feasible in adolescents [36]. We chose a dosing schedule of five 25-minute sessions per week given our experience with cognitive rehabilitation in children and prior research demonstrating that shorter, more frequent (as opposed to longer, less frequent) training sessions would be most feasible in youth and would result in the most behavioral change [36-38]. Each participant was assigned a training coach on the study team who worked with them to create a personalized training schedule that included 5 training sessions across 5 unique days in a given week and one 15-minute videoconference check-in with the training coach each week. During weekly check-ins, participants engaged in a brief videoconference with their training coach, who reviewed their progress in the training program, addressed any barriers to adherence and engagement, and adjusted their training schedule for the following week if needed (eg, if the participant had a family event that weekend and wanted to complete training on Saturday instead of Sunday). Participants were compensated for their time in completing assigned training each week, and their compensation earnings were reviewed each week during weekly check-ins (see Ethical Considerations). Dose adherence (percentage of assigned levels completed) and regimen adherence (percentage of scheduled training sessions completed) were examined as measures of feasibility.

Following completion of the training, participants provided responses to questions probing program acceptability (see Multimedia Appendix 1). The acceptability survey was adapted from existing measures [37,39] and consisted of 15 statements on a 7-point Likert scale with the following response options: 1, strongly disagree; 2, disagree; 3, somewhat disagree; 4, neither agree nor disagree; 5, somewhat agree; 6, agree; and 7, strongly agree. Acceptability questions were grouped into categories representing usability, adoptability, and perceived benefit. Acceptability ratings were averaged for each participant by domain and for overall acceptability, which were then used to calculate group-based averages. Group-based average scores greater than 4 were interpreted as more positive than neutral views toward the training program, suggestive of adequate acceptability.

Participants took the Double Decision Assessment [40] pre- and posttraining to quantify the magnitude of change in their visual processing efficiency. The Double Decision Assessment is a computerized, adaptive, train-to-task assessment that mimics a level of UFOV training. Threshold scores on the Double Decision Assessment and on UFOV training levels indicate the fastest stimulus presentation duration at which participants are able to accurately identify and locate stimuli presented simultaneously in the center and at the periphery of the visual field among distractors, with an 80% threshold determined via an adaptive staircase algorithm. Lower threshold scores indicate faster visual processing and better performance. Magnitude of change in visual processing efficiency was preliminarily evaluated by calculating the mean difference in performance on the Double Decision Assessment from pre- to postintervention. Paired Cohen *d* was calculated as a measure of effect size. Statistical analyses and data visualization were conducted using R software, version 4.5.1 (R Foundation for Statistical Computing) [41]. There were no missing data.

### Ethical Considerations

This study was approved by the Johns Hopkins University School of Medicine Institutional Review Board (IRB00374143). Prior to participation, informed consent was obtained from legal guardians of participants aged 17 years and younger and from participants who were aged 18 years. Protective measures were taken to safeguard participant information, including the use of deidentified research accounts for accessing the BrainHQ portal. Participants were compensated US $10 per 10-level training session completed. In addition, participants were compensated for total levels completed during the week (US $0.50 per level up to US $25 per week). This allowed participants who did not complete a full session to be compensated for their time.

## Results

### Feasibility

**Figure 2.**
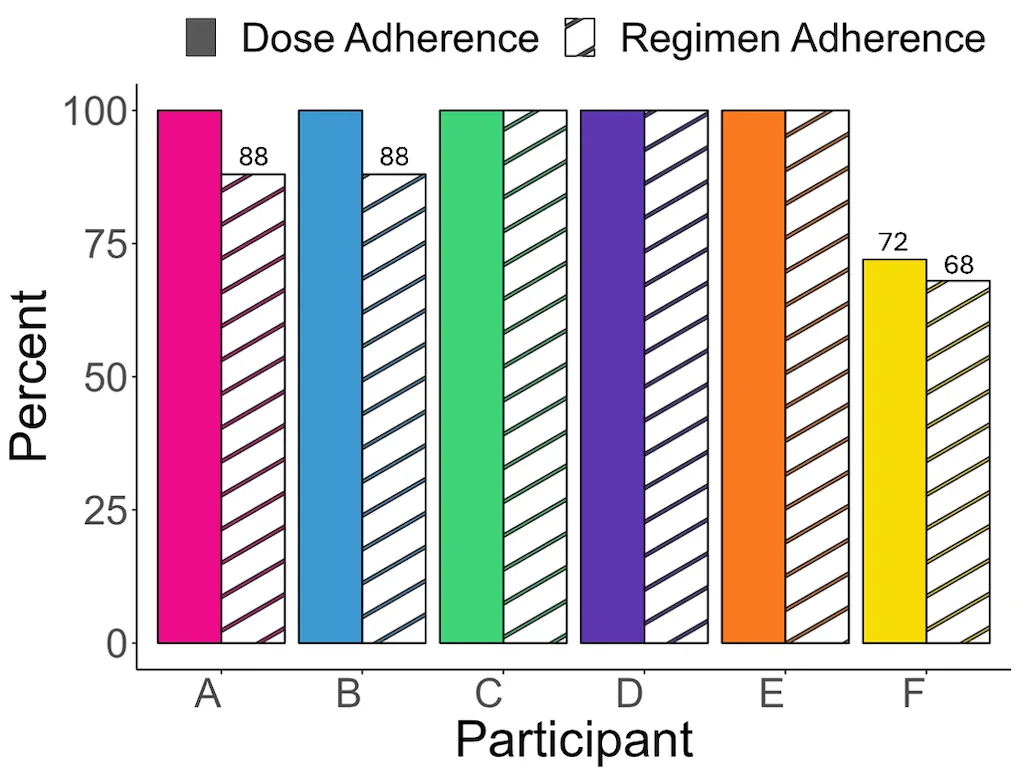
Dose adherence and regimen adherence for each participant.

Participants trained for a mean of 9.69 (SD 1.49) hours in total. Mean dose adherence was 95.33% (SD 11.43%) and mean regimen adherence was 90.67% (SD 12.56%; Figure 2). Participants’ mean earnings were US $345.83 (SD 44.54, range, US $260-$375) at completion of training.

### Acceptability

The mean overall acceptability rating was 5.47 (SD 0.4). Mean usability, perceived benefit, and adoptability ratings were 5.69 (SD 0.43), 5.36 (SD 0.69), and 5.17 (SD 0.61), respectively; see Multimedia Appendix 1 for item-level averages across all participants. Notably, all participants indicated that they somewhat agreed, agreed, or strongly agreed with statements asserting that they felt satisfied during training (usability), that the program could help with driving performance and safety (perceived benefit), and that they would recommend the training to others (adoptability). All participants but one indicated that the training program seemed like it could help athletes participate in sports more safely.

### Magnitude of Change in Visual Processing Efficiency

**Figure 3.**
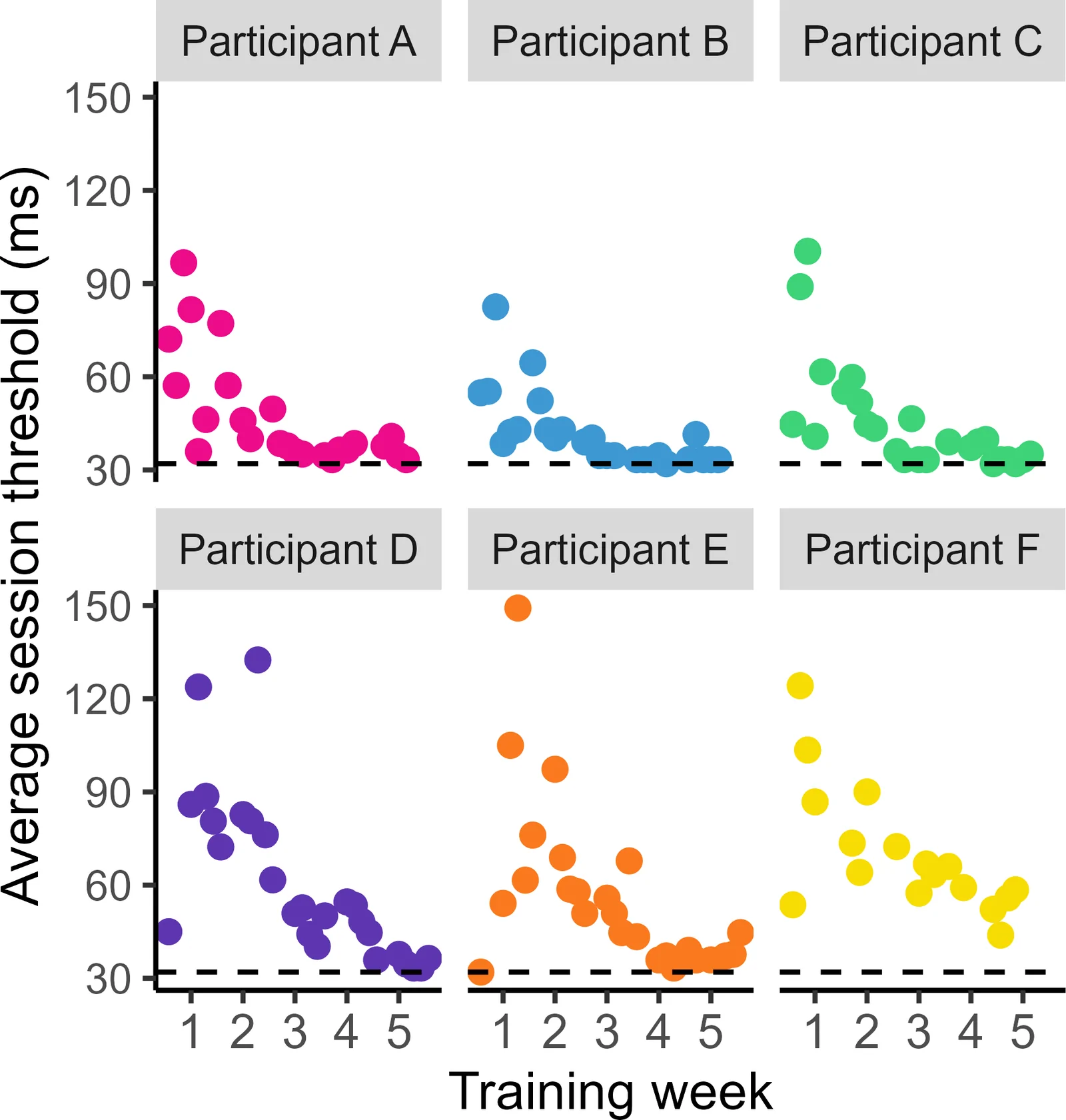
Progress on useful field of view (UFOV) training levels throughout the course of training (5 weeks) for each participant. Threshold scores on individual levels were averaged within each training session. Dashed lines represent the best possible threshold score on any given level (32 ms). Lower threshold scores indicate faster visual processing efficiency and better performance.

Performance on training levels improved throughout training for all participants; within-session average threshold scores on UFOV training levels approached ceiling (32 ms) for each participant by the end of the 5-week program (Figure 3).

**Figure 4.**
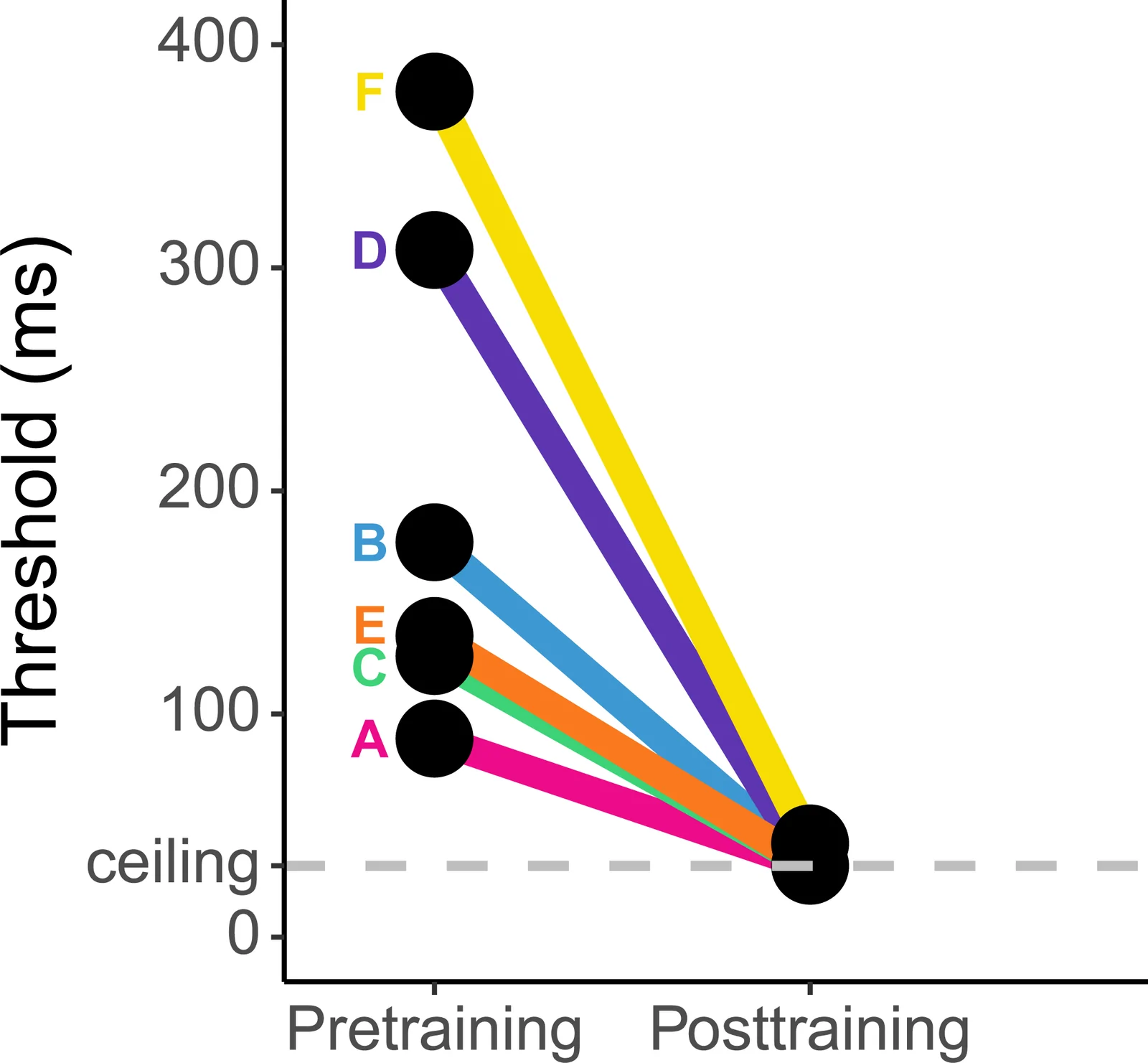
Scores on the Double Decision Assessment pretraining and posttraining for each participant. Lower threshold scores reflect more efficient visual processing. Participants D and F had a history of concussion (>1 y after clinical recovery).

On the Double Decision Assessment, all participants improved from pre- to posttraining, with all but 1 participant exhibiting the best possible score on the measure after training (Figure 4). The mean difference in threshold score from pre- to posttraining was 168.67 (SD 112.05) ms (Cohen *d*=1.51). The 2 participants with a history of concussion (participants D and F in Figure 4) demonstrated the highest threshold scores prior to training and the greatest improvement from pre- to posttraining (mean difference 306.50, SD 43.13 ms).

## Discussion

Preliminary evidence shows that a 5-week UFOV training program is feasible for adolescents with the coaching support and compensation provided in this research setting. This training program can be completed at home or elsewhere and is commercially available, with strong potential for scalability. Although compensating adolescents for engaging in training may not be feasible at scale, we expect that once efficacy is better established, anticipated gains and program benefits will be enough incentive for engagement. Additionally, prior work has demonstrated the value of incorporating a training coach as part of remote cognitive training programs [41,42], which we expect was helpful in supporting engagement and adherence in this pilot study. Training coaches worked with participants to establish personalized training schedules, which gave participants flexibility and allowed them to optimally schedule their training amid other engagements (full-time jobs or internships, school, club sports, or other extracurriculars) while maintaining the fidelity of the program and receiving the target dosage. Of note, we deliberately enrolled participants during the summer months, as we expected that training would be most feasible for youth athletes during months when school was not in session. However, 3 participants started school toward the end of the training program, with 2 of the 3 still maintaining strong adherence during this time. Future work is needed to determine if this training program would be optimally deployed during a specific time of year or if the intervention or coaching sessions should ideally be delivered in an individual versus group setting [43].

Our early findings also suggest a high degree of intervention acceptability. On average, participants indicated positive views toward the program in terms of how enjoyable and easy to use it was, that it seemed beneficial, and that they would use the program outside of the study or recommend it to others. Though meaningful, these results are limited to participant responses to a survey, which are subject to bias and may not reflect participants’ true views of the intervention. Future work incorporating qualitative approaches may also be informative and could shed light on further opportunities to enhance acceptance of the intervention.

We observed improvement in visual processing efficiency from pre- to posttraining in this initial proof-of-concept study. The presence of posttraining ceiling scores suggests potential measurement constraints of the Double Decision Assessment in high-performing adolescents, which may limit sensitivity to additional gains. Future randomized trials should evaluate whether the skills adolescents gain from training result in enhanced performance on vision-dependent tasks and transfer to untrained tasks and real-world safety outcomes. Given the critical role of visual processing in sport performance, on-field reaction time, and collision avoidance, we theorize that improving visual processing with UFOV training may reduce sport-related injuries among adolescent athletes. However, it remains unknown whether improvements in computerized threshold-based tasks translate to complex, dynamic sport environments that require anticipation, motor coordination, and multisensory integration. Data on the longevity of skill transfer in adults suggests that if efficacious, benefits may persist throughout the sports season and beyond [17,19,25-30]. Mounting evidence has demonstrated that adolescent athletes with a history of concussion are at increased risk for sustaining repeat concussions or new musculoskeletal injuries, even after they are considered fully recovered by medical providers [44-47]. The current study included 2 adolescents with a prior history of concussion who were able to complete the intervention; future studies designed to evaluate the efficacy of UFOV training may consider enrolling adolescent athletes who are returning to sport after concussion and are therefore at heightened risk for repeat injuries.

The current study represents an important first step in evaluating UFOV training as a feasible tool to enhance visual processing in adolescents. However, the authors acknowledge that this preliminary study was significantly limited in terms of its small sample size and associated threats to generalizability. While we were able to quantify pre-post change in visual processing efficiency, a follow-up randomized controlled trial will be needed to evaluate the efficacy of UFOV, ideally relative to an active control intervention.

To our knowledge, this is the first study to implement and report on UFOV cognitive training in adolescents. Our preliminary results demonstrate that it is feasible to utilize this intervention in adolescents and not only in adult populations. However, the supports included in this study do not reflect real-world use, and future work will be needed to evaluate feasibility in larger studies and in other contexts (eg, team-based environments with additional structure but without support or compensation from the research team). Future work investigating the cognitive and real-world benefits of UFOV training in adolescents could lead to meaningful safety improvements for athletes as well as other adolescent populations who would benefit from enhanced visual processing abilities, such as adolescents learning to drive.

## Supplementary material

10.2196/93778 Multimedia Appendix 1 Acceptability survey results (N=6).

## Abbreviations

UFOV: useful field of view
