# Supplementary material for: Feasibility and Acceptability of Useful Field of View Cognitive Training in Adolescent Athletes: Pilot Pre-Post Study
Source: JMIR Form Res. 2026 Sep 11;10:e93778. doi: 10.2196/93778 (PMC13566078; doi:10.2196/93778)
Supplement: Supplementary file 1 [file formative-v10-e93778-s001.docx]

Acceptability survey results (n=6).

| Domain | Item(s) | Statement | Mean (SD) |
| --- | --- | --- | --- |
|  | 1 | I enjoyed the training program | 5.50 (0.84) |
|  | 2 | I felt satisfied after training sessions | 6.00  (0.89) |
|  | 3 | The program was easy to use | 6.33 (0.52) |
|  | 4 | The program was easy to initiate each day | 6.17 (0.75) |
|  | 5 | The program easily fit in my daily schedule | 4.83 (1.72) |
|  | 6 | The training sessions passed quickly | 5.33 (0.52) |
| Usability | 1-6 |  | 5.69 (0.43) |
|  | 7 | The training seemed like it could improve my visual ability | 5.50 (0.84) |
|  | 8 | The program seemed like it could help me think or react more quickly | 5.83 (0.98) |
|  | 9 | The program seemed like it could help me focus my attention on two things at once | 4.83 (1.17) |
|  | 10 | The training seemed like it could improve sport or athletic performance | 5.17 (1.17) |
|  | 11 | The program seemed like it could help athletes participate in sport more safely | 4.67 (1.03) |
|  | 12 | The program seemed like it could help driving performance | 5.83 (0.75) |
|  | 13 | The program seemed like it could help drivers be safer on the road | 5.67 (0.52) |
| Perceived Benefit | 7-13 |  | 5.36 (0.69) |
|  | 14 | I would use this training outside of the study if I knew it would be helpful to me | 4.83 (1.17) |
|  | 15 | I would recommend this training to others | 5.50 (0.55) |
| Adoptability | 14-15 |  | 5.17 (0.61) |
| Overall Acceptability | 1-15 |  | 5.47 (0.83) |

NOTE: Average acceptability ratings are presented for each item across all participants. For domain and overall acceptability averages, these were computed by obtaining participant-level averages within each acceptability domain and then averaging them across all participants.
